# The Performance of Enhanced Liver Fibrosis (ELF) Test for the Staging of Liver Fibrosis: A Meta-Analysis

**DOI:** 10.1371/journal.pone.0092772

**Published:** 2014-04-15

**Authors:** Qingsong Xie, Xiaohu Zhou, Pengfei Huang, Jianfeng Wei, Weilin Wang, Shusen Zheng

**Affiliations:** 1 Division of Hepatobilitary and Pancreatic Surgery, Department of Surgery, First Affiliated Hospital, School of Medicine, Zhejiang University, Hangzhou, Zhejiang Province, China; 2 Key Laboratory of Combined Multi- Organ Transplantation, Ministry of Public Health, Zhejiang Province, Hangzhou, China; 3 Key laboratory of Organ Transplantation, Zhejiang Province, Hangzhou, China; The University of Hong Kong, Hong Kong

## Abstract

**Background:**

The enhanced liver fibrosis test (ELF) has been shown to accurately predict significant liver fibrosis in several liver diseases.

**Aims:**

To perform a meta-analysis to assess the performance of the ELF test for the assessment of liver fibrosis.

**Study:**

Electronic and manual searches were performed to identify studies of the ELF test. After methodological quality assessment and data extraction, pooled estimates of the sensitivity, specificity, area under the receiver operating characteristic curve (AUROC), positive likelihood ratio (PLR), negative likelihood ratio (NLR), diagnostic odds ratio (DOR) and summary receiver operating characteristics (sROC) were assessed systematically. The extent of heterogeneity and reasons for it were assessed.

**Results:**

Nine studies were identified for analysis. The pooled sensitivity, specificity, positive LR, negative LR, and DOR values of ELF test, for assessment of significant liver fibrosis, were 83% (95% CI = 0.80–0.86), 73% (95% CI = 0.69–0.77), 4.00 (95% CI = 2.50–6.39), 0.24 (95% CI = 0.17–0.34), and 16.10 (95% CI = 8.27–31.34), respectively; and, for evaluation of severe liver fibrosis, were 78% (95% CI = 0.74–0.81), 76% (95% CI = 0.73–0.78), 4.39 (95% CI = 2.76–6.97), 0.27 (95% CI = 0.16–0.46), and 16.01 (95% CI: 7.15–35.82), respectively; and, for estimation of cirrhosis, were 80% (95% CI = 0.75–0.85), 71% (95% CI = 0.68–0.74), 3.13 (95% CI = 2.01–4.87), 0.29 (95% CI = 0.19–0.44), and 14.09 (95% CI: 5.43–36.59), respectively.

**Conclusions:**

The ELF test shows good performance and considerable diagnostic value for the prediction of histological fibrosis stage.

## Introduction

Liver fibrosis is a consequence of various chronic liver diseases, often caused by viruses, alcohol, and fat deposition, and can result in liver cirrhosis. Cirrhosis is the main cause of morbidity and mortality in chronic liver disease, but is often asymptomatic until the synthetic and filtering functions of the liver are finally compromised or portal hypertension develops. In addition, for chronic viral hepatitis, the degree of liver fibrosis is an important parameter for decisions on antiviral therapy [1], so the early detection of fibrosis progression and the development of cirrhosis are important in the management of patients with chronic liver disease.

Presently, a liver biopsy remains the reference standard for evaluating liver fibrosis. However, it is limited by sampling error and the risk of complications [2], [3]. Intra- and interobserver variability may lead to misinterpretation of the fibrosis stage [4]–[7]. One reason for the difficulty in correctly assessing the fibrosis stages may be simply that a biopsy specimen represents only 1/50,000^th^ of the total liver mass [2]. Even with adequate biopsy samples (≥15 mm in length with five or more portal tracts), cirrhosis can be understaged in 10–30% of cases [8]. Moreover, it is usually difficult to undertake biopsies on a repeated basis, because of their invasive nature and complications, such as pain and bleeding.

Thus, much attention has been focused on the development of non-invasive methods, including radiological and biochemical tests, to detect liver fibrosis. Transient elastography for assessing liver stiffness has become available for the evaluation of liver fibrosis as a rapid, non-invasive method. However, this technique is cost-intensive and its availability is largely limited to specialist liver centres. Moreover, liver stiffness measurements can be difficult or impossible in obese patients, in those with narrow intercostal space, and in patients with ascites [9], and a failure rate up to 18.9% has been reported [10].

Alternative method assessing the degree of liver fibrosis focused on serum biomarkers. The combined use of three serum biomarkers of hyaluronic acid (HA) [11], which is a component of the extracellular matrix (ECM) and is primarily cleared from the bloodstream by the hepatic sinusoids, tissue inhibitors of metalloproteinases (TIMP-1) [12]–[16] inhibiting the activities of matrix metalloproteinases (MMPs) and amino-terminal propeptide of procollagen type III (PIIINP) [17]–[20] reflecting collagen synthesis at the site of disease has recently been proposed for the detection of fibrosis. In clinical practice, serum samples were analysed for levels of HA, TIMP-1 and PIIINP. Results were entered into the established algorithm and expressed as discriminant scores. This simplified version of panel was called enhanced liver fibrosis (ELF) score. In other word, a higher concentration of individual biomarkers leads to a higher ELF score and indicates a greater likelihood of more severe fibrosis. The ELF test has several strengths such as better automaticity, high reproducibility, less invasiveness and proven considerable diagnostic performance in the assessment of the degree of liver fibrosis [21]–[23]. The ELF test has received the Conformité Européénne mark in May 2007 [24].

The aim of this study was to perform a meta-analysis to evaluate the diagnostic accuracy of ELF, with histopathology as a reference standard.

## Methods

### Search Strategy

A computerised search was performed in PubMed/Medline, EMBASE, the Cochrane Library, and Google Scholar to identify relevant articles published from 2003 to 2013. The literature search was performed with the following terms: cirrhosis, liver fibrosis, and enhanced liver fibrosis test or ELF test. The research was limited to articles concerning humans with an abstract in English. The complete search yielded 260 articles from databases.

### Study Selection and Quality Assessment

Two reviewers (W-L.W. and Q-S.X.) read the titles and abstracts of original articles that addressed the diagnostic accuracy of ELF for staging liver fibrosis in humans to select potentially relevant articles. All of the selected articles were collected and reviewed independently by the same reviewers to determine their eligibility for detailed analysis. The inclusion criteria were as follows: patients with suspected cirrhosis, ELF scores as the index test, defined optimal cut-off values or a threshold of ELF, histopathology as the reference test, and raw data (i.e., true-positive (TP), false-positive (FP), true-negative (TN), and false-negative (FN) results could be found or calculated). Exclusion criteria were duplicate publication (based on the same primary study) and sample size of less than 20. Disagreements between the two reviewers regarding study inclusion were resolved by consensus after a face-to-face discussion. Investigators in the primary research were approached for additional information as necessary.

The methodological quality of each study was assessed using a checklist based on the Quality Assessment for Studies of Diagnostic Accuracy (QUADAS) tool [25], which enables reviewers to evaluate the quality of studies, especially investigations of diagnostic accuracy [26], [27].

### Data Extraction

Data were extracted from primary studies by the two reviewers (W-L.W. and Q-S.X.) independently. In cases of discrepancies between the first two reviewers, a senior surgeon (S-S.Z.), with more than 20 years of experience in hepatic disease, was consulted and a consensus was reached. We defined significant fibrosis as a fibrosis stage ≥2 for studies using grading systems with five stages (F0–F4; i.e., the METAVIR, Brunt, Batts-Ludwig systems) or as a fibrosis stage ≥3 for studies using the Ishak scoring system (S0–S6). For grading systems using five stages or the Ishak scoring system, severe fibrosis was defined as a fibrosis stage ≥3 or ≥4, and cirrhosis was defined as a fibrosis stage = 4 or ≥5, respectively [28]. We extracted available data on TPs, FNs, FPs, and TNs for staging liver fibrosis to construct a 2×2 contingency table.

### Data Synthesis and Statistical Analysis

From the extracted data, arranged in 2×2 contingency tables, we computed sensitivity, specificity, and diagnostic odds ratios (DORS) to estimate the diagnostic performance of each test modality to assess each stage of liver fibrosis. All statistics are reported as point values with 95% confidence intervals (CIs). Sensitivity was defined by the TP rate and was calculated as TP/(TP+FN). Specificity was defined by the TN rate and was calculated as TN/(FP+TN). The DOR is a single overall indicator of diagnostic performance and is the ratio of the odds of positivity in disease subjects relative to the odds of positivity in non-diseased subjects [29]. The DOR was calculated as (TP×TN)/(FP×FN).

The performance was summarised using a bivariate binomial model [30]. This model assumed a binomial distribution for the number of patients with TP and TN results and allowed the inclusion of covariates and random effects. The inherent association between sensitivity and specificity was modelled in a bivariate normal distribution by assuming random effects [31].

The heterogeneity of all diagnostic test parameters was evaluated initially with a graphic examination of forest plots for each parameter. A statistical assessment was then made of the inconsistency index (I^2^). The I^2^ statistic is defined as the percentage of variability due to heterogeneity beyond that from chance; values greater than 50% represent the possibility of substantial heterogeneity. The pooled summary statistics for the sensitivities, specificities, likelihood ratios, and diagnostic odds ratios of the individual studies are reported.

Summary receiver operating characteristic (SROC) curves were also constructed to express the test parameter results as the diagnostic odds ratios. These curves were also used to assess the presence of a diagnostic threshold (cut-off) bias as a cause of between-study heterogeneity. Analyses were performed using the Meta-Disc 1.4 statistical software (Unit of the Clinical Biostatistics team of the Ramón y Cajal Hospital in Madrid, Spain).

## Results

### Study Characteristics

**Figure 1:**
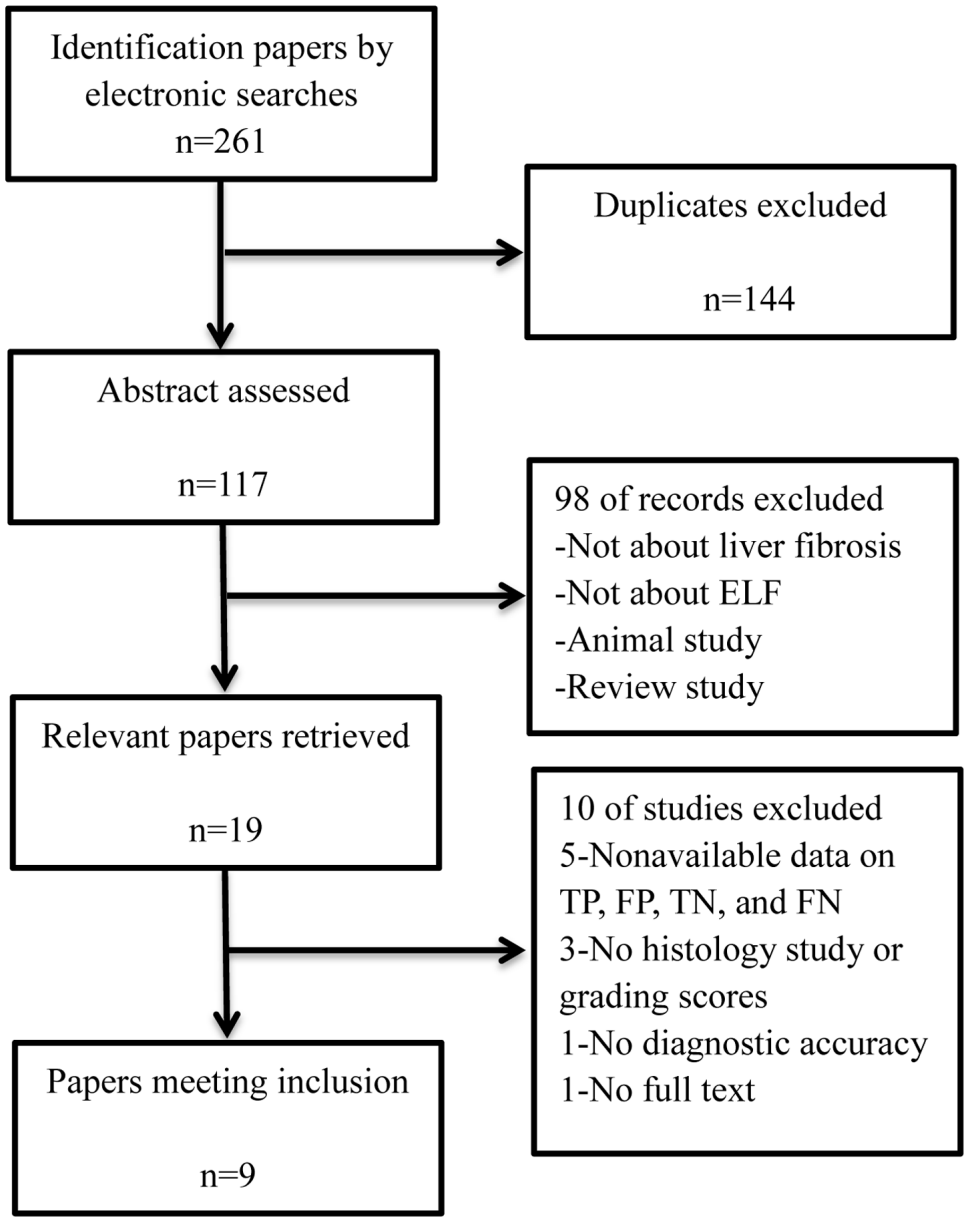
Flowchart for the literature search.

Figure 1 depicts the flow of our search results. In total, 261 studies were identified using electronic searches. Without duplicates, 117 abstracts were assessed. Of them, 19 seemed relevant and the full studies were assessed. Ultimately, nine investigations were identified for inclusion in this study [32]–[40]. Quality assessment scores for the diagnostic studies were above 10 of the 14 QUADAS items describing methodological quality.

The nine studies evaluated involved 1826 patients from Asian and European medical centres. In four trials, the disease spectrum was restricted to chronic viral hepatitis, in one trial to non-alcoholic fatty liver disease (NAFLD), and in four trials, there was no restriction (Table 1).

**Table 1:** Characteristic of patients of included studies.

Study	Date	Country	Range timeof study	PatientsN	Male (%)	Age (mean±SD)	Disease spectrum	Fibrosis stage (%)
**Guha, I.N. ** ***et al***	2008	UK	2003–2006	192	64	50±13	CHC, CHB, PBC	F0∶41, F1∶19, F2∶17, F3∶13, F4∶10
**Nobili, V. ** ***et al***	2009	UK	2004–2006	112	56.2	14.1±3.7	NAFLD	F0∶33, F1∶51.8,F2∶8, F3–4∶7.2
**Friedrich-Rust, M. ** ***et al***	2010	Germany	2005–2008	74	41.9	50±13	CHC, CHB, PBC	F0∶5.4,F1∶28.4,F2∶24.3,F3∶27, F4∶14.9
**Parkes, J. ** ***et al***	2011	UK	1999–2001	347	61	43(19–75)§	CHC	F0∶13.3,F1∶26.8,F2∶31.1,F3–4∶28.8
**Kim, B.K. ** ***et al***	2012	Korea	2010–1011	170	60	45.3±15.1	CHB	F0∶5.9,F1∶22.9,F2∶21.2,F3∶22.4, F4∶27.6
**Wahl, K ** ***et al***	2012	Germany	NR	102	52	46.6±1.3	VH, AIH, Wilson’s diseases, NALFLD, others	F0–1∶66.7,F2–4∶22.5,F5–6∶10.8
**Guechot, J. ** ***et al***	2012	France	2007–2008	512	59.8	50(18–79)§	CHB, ALD, CHC, other	F0∶6.6,F1∶45.1,F2∶18,F3∶15.4, F4∶14.9
**Lichtinghagen, R. ** ***et al***	2013	Germany	NR	NR	NR	NR	CHC	F0∶26.6,F1–2∶24.1,F3–4∶12.7,F5–6∶36.7
**Wong GL**	2014	Hong Kong	2006–2009	238	80	50±11	CHB	F0∶6, F1∶24, F2∶29, F3∶16, F4∶24

§ Values are median age and range of age of patients. Abbreviations: NR, not reported; CHC, chronic hepatitis C; CHB, chronic hepatitis B; PBC, primary biliary cirrhosis; NAFLD, Non-Alcoholic Fatty Liver Disease; VH, viral hepatitis; AIH, autoimmune hepatitis; ALD, alcoholic liver disease.

Five studies reported quality criteria for liver biopsy specimens [32], [34], [36]–[38], and three investigations reported a minimum length of 15 mm [33], [35], [39]. The Ishak histological scoring system was used in three studies [35], [38], [39], the National Institute of Diabetes and Digestive and Kidney Diseases system was used in one study [33], the modified Brunt system was used in one study [32], the Batts-Ludwig system was used in one study [37], and the METAVIR system was used in four studies [34]–[36], [40] (Table 2). Moreover, there are four studies [34], [37], [38], [40] comparing the performance of ELF test with transient elastograhpy (TE) for staging liver fibrosis (Table 3).

**Table 2:** Test characteristics and histological scoring system of included studies of ELF for the assessment of fibrosis.

Study	Referencetest	Histologicalscoringsystem	Liver biopsylength(mm)	FibrosisStage	Cut-offValue	Sensitivity	Specificity	Positive predictivevalue	Negative predictiveValue	AUROCvalue(95%CI)
**Guha, I.N. ** ***et al***	liver biopsy	NIDDKD	NR	≥F2	−0.11	0.7	0.8	0.7	0.8	0.82(0.75–0.88)
				≥F3	0.357§	0.8	0.9	0.71	0.94	0.9(0.84–0.96)
**Nobili, V. ** ***et al***	liver biopsy	Modified Brunt	20.7±2.3	≥F2	10.18	0.94	0.93	0.7	0.99	0.98(0.96–1)
				≥F3	10.51	1	0.98	0.8	1	0.99(0.97–1)
**Friedrich-Rust, M. ** ***et al***	liver biopsy	METAVIR	22.3±9.3	≥F2	9.78	0.78	0.8	0.88	0.65	0.78(0.67–0.89)
				≥F3	10.22	0.74	0.7	0.64	0.79	0.79(0.67–0.91)
				= F4	10.31	0.91	0.62	0.29	0.98	0.92(0.83–1)
**Parkes, J. ** ***et al***	liver biopsy	METAVIR/Ishak	NR	= F5–6	10.48§	0.62	0.89	0.73	0.83	0.86(0.83–0.89)
**Kim, B.K. ** ***et al***	liver biopsy	Batts-Ludwig	21.3±0.7	≥F2	8.5	0.86	0.857	0.937	0.712	0.901(0.849–0.953)
				≥F3	9.4	0.835	0.777	0.789	0.825	0.86(0.805–0.915)
				= F4	10.1	0.702	0.789	0.559	0.874	0.862(0.809–0.915)
**Wahl, K. ** ***et al***	liver biopsy	Ishak	18.9±2.1	≥F2	8.99	0.86	0.7	NR	NR	0.87(0.78–0.96)
				= F5–6	9.39	1	0.77	NR	NR	0.93(0.88–0.99)
**Guechot, J. ** ***et al***	liver biopsy	METAVIR	25.1±8.8	≥F2	9	0.86	0.62	0.8	0.7	0.78(0.74–0.82)
				≥F3	9.33	0.9	0.63	0.73	0.85	0.82(0.78–0.86)
				= F4	9.35	0.83	0.75	0.44	0.95	0.85(0.81–0.9)
**Lichtinghagen, R. ** ***et al***	liver biopsy	Ishak	NR	≥F3	9.8	0.846	0.75	NR	NR	0.95(NR)
				= F5–6	11.3	0.828	0.96	NR	NR	0.9(NR)
**Wong GL. ** ***et*** § ***al***	liver biopsy	METAVIR	NR	≥F3	9.8	0.62	0.66	0.55	0.72	0.69(0.63–0.75)
				= F4	9.5	0.78	0.47	0.31	0.88	0.68(0.61–0.75)

§ Values are diagnostic threshold. Abbreviations: NR, not reported; NIDDKD, National Institute of Diabetes and Digestive and Kidney Diseases.

**Table 3:** Characteristics and performance of three studies comparing ELF test with transient elastography (TE).

Study	PatientN	Fibrosisstage (%)	Methods	FibrosisStage	Cut-offValue	Sensitivity	Specificity	Positivepredictivevalue	NegativepredictiveValue	AUROCvalue(95%CI)
**Friedrich-Rust,** **M. ** ***et al*** ** 2010**	102	F0∶5.4,F1∶28.4,F2∶24.3,F3∶27,F4∶14.9	ELF	≥F2	9.78	0.78	0.8	0.88	0.65	0.78(0.67–0.89)
				≥F3	10.22	0.74	0.7	0.64	0.79	0.79(0.67–0.91)
				= F4	10.31	0.91	0.62	0.29	0.98	0.92(0.83–1)
			TE	≥F2	7.2 kPa	0.64	0.76	0.85	0.5	0.8(0.69–0.91)
				≥F3	12.5 kPa	0.5	0.87	0.74	0.76	0.66(0.51–0.82)
				= F4	17.6 kPa	0.82	0.91	0.64	0.78	0.94(0.86–1)
**Kim, B.K.** ***et al*** ** 2012**	170	F0∶5.9,F1∶22.9,F2∶21.2,F3∶22.4,F4∶27.6	ELF	≥F2	8.5	0.86	0.857	0.937	0.712	0.901(0.849–0.953)
				≥F3	9.4	0.835	0.777	0.789	0.825	0.86(0.805–0.915)
				= F4	10.1	0.702	0.789	0.559	0.874	0.862(0.809–0.915)
			TE	≥F2	8 kPa	0.777	0.959	9.979	0.635	0.937(0.903–0.971)
				≥F3	10.1 kPa	0.906	0.965	0.962	0.911	0.956(0.929–0.983)
				= F4	14 kPa	0.872	0.91	0.788	0.949	0.963(0.937–0.989)
**Wahl, K** ***et al*** ** 2012**	102	F0–1∶66.7,F2–4∶22.5,F5–6∶10.8	ELF	≥F2	8.99	0.86	0.7	NR	NR	0.87(0.78–0.96)
				≥F5	9.39	1	0.77	NR	NR	0.93(0.88–0.99)
			TE	≥F2	8.5 kPa	0.86	0.73	NR	NR	0.92(0.85–0.98)
				≥F5	17.45	0.91	1	NR	NR	0.95(0.87–1)
**Wong GL** ***et§al*** ** 2014**	238	F0∶6,F1∶24,F2∶29,F3∶16, F4∶24	ELF	≥F3	9.8	0.62	0.66	0.55	0.72	0.69(0.63–0.75)
				= F4	9.5	0.78	0.47	0.31	0.88	0.68(0.61–0.75)
			TE	≥F3	9 kPa	0.64	0.84	0.69	0.76	0.83(0.76–0.91)
				= F4	10 kPa	0.78	0.81	0.56	0.93	0.9(0.84–0.96)

Abbreviations: ELF, enhanced liver fibrosis; TE, transient elastography.

The demographic and clinical features of the patients in the studies analysed are listed in Table 1. The median sample size of the studies assessing the presence of significant liver fibrosis was 141 (range, 74–512) [32]–[34], [36], [37], [39], the median sample size of the studies assessing the presence of severe liver fibrosis was 181 (range, 74–512) [32]–[37], [40], and the median sample size of the studies assessing the presence of cirrhosis was 102 (range, 74–512) [34], [36]–[39], [40].

In the studies analysed, the median proportion of individuals with cirrhosis was 14.9% (range, 10–36.7%). The diagnostic cut-off values ranged from 8.5 to 10.18 for significant liver fibrosis [32]–[34], [36], [37], [39], from 9.33 to 10.51 for severe liver fibrosis [32]–[37], [40], and from 9.35 to 11.3 [32], [34]–[37], [40] for cirrhosis.

### Diagnostic Threshold Bias and Meta-Regression Assessment

**Figure 2:**
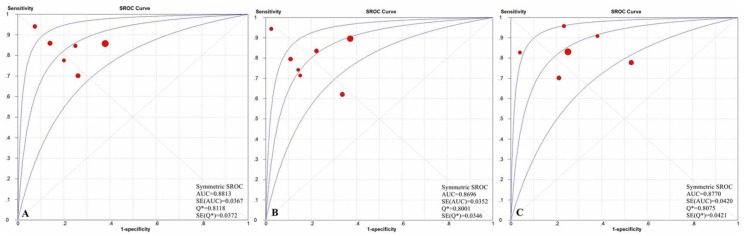
The SROC curves for the studies examining ELF test versus liver biopsy for the assessment of (A) significant liver fibrosis, (B) severe liver fibrosis and (C) cirrhosis.

To assess the diagnostic threshold (cut-off) bias as a cause of heterogeneity in test performance, we prepared an ROC plot of the sensitivity versus 1-the specificity. Among the six primary studies providing data for the detection of significant liver fibrosis, the diagnostic threshold (cut-off) yielded an area under receiver operating characteristic (AUROC) of 0.8813, among another seven primary studies providing data for the assessment of severe liver fibrosis, the diagnostic threshold (cut-off) yielded an AUROC of 0.8696, among another six primary studies providing data for the prediction of cirrhosis, the diagnostic threshold (cut-off) yielded an AUROC of 0.8770, and they all revealed evidence supporting the diagnostic threshold (cut-off) bias as a major cause of heterogeneity(Fig. 2).

### Summary Estimates of Primary Studies

**Figure 3:**
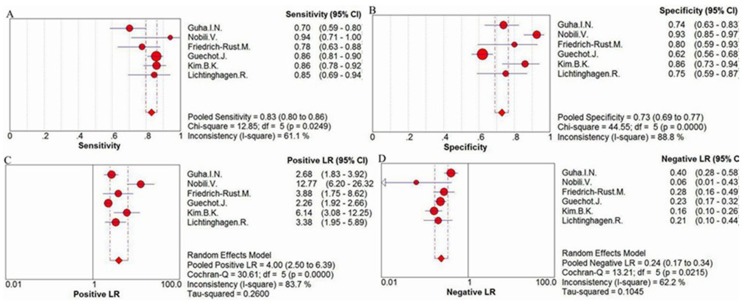
Forest plot and meta-analysis of studies assessing (A) the sensitivity, (B) the specificity, (C) the positive LR and (D) the negative LR of ELF test versus biopsy for the detection of significant liver fibrosis.

**Figure 4:**
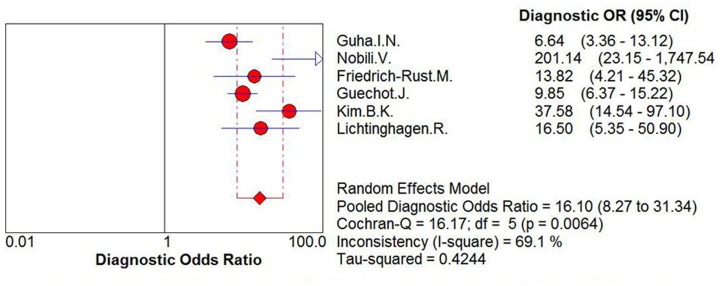
Forest plot and meta-analysis of studies appraising the diagnostic odds ratio of ELF test versus liver biopsy for the assessment of significant liver fibrosis.

For the assessment of significant liver fibrosis, the sensitivity values of ELF ranged from 70% to 94%, and the pooled sensitivity value was 83% (95% CI = 0.8–0.86, P = 0.0249, I^2^ = 61.1%). The specificity values ranged from 62% to 93%, and the pooled specificity value was 73% (95% CI = 0.69–0.77, P<0.0001, I^2^ = 88.8%). The pooled positive likelihood ratio was 4.00 (95% CI = 2.03–6.39, P<0.0001, I^2^ = 83.7), and the pooled negative likelihood ratio was 0.24 (95% CI = 0.17–0.34, P = 0.0215, I^2^ = 62.2%) (Fig. 3) The summary diagnostic odds ratio was 16.10 (95% CI = 8.27–31.34, P = 0.0064, I^2^ = 69.1%) (Fig. 4).

**Figure 5:**
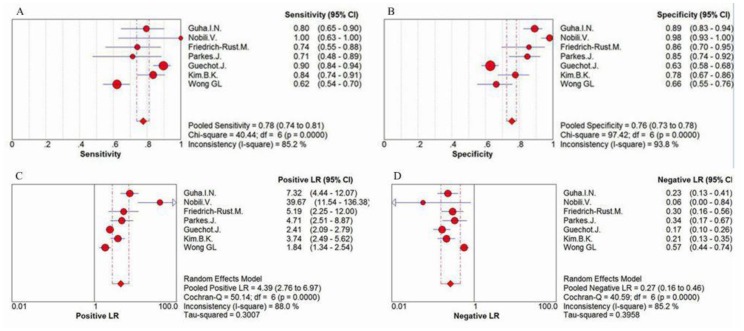
Forest plot and meta-analysis of studies estimating (A) the sensitivity, (B) the specificity, (C) the positive LR and (D) the negative LR of ELF test versus liver biopsy for the detection of severe liver fibrosis.

**Figure 6:**
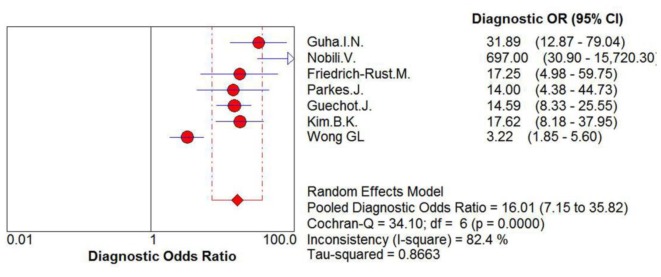
Forest plot and meta-analysis of studies assessing the diagnostic odds ratio of ELF test versus liver biopsy for the measurement of severe liver fibrosis.

For the prediction of severe liver fibrosis, the sensitivity values of ELF test ranged from 62% to 100%, and the pooled sensitivity value was 78% (95% CI = 0.74–0.81, P<0.0001, I^2^ = 85.2%). The specificity values ranged from 66% to 98%, and the pooled specificity value was 76% (95% CI = 0.73–0.78, P<0.0001, I^2^ = 93.8%). The pooled positive likelihood ratio was 4.39 (95% CI = 2.76–6.97, P<0.0001, I^2^ = 88.0%), and the pooled negative likelihood ratio was 0.27 (95% CI = 0.16–0.46, P<0.0001, I^2^ = 85.2%) (Fig. 5) The summary diagnostic odds ratio was 16.01 (95% CI = 7.15–35.82, P<0.0001, I^2^ = 82.4%) (Fig. 6).

**Figure 7:**
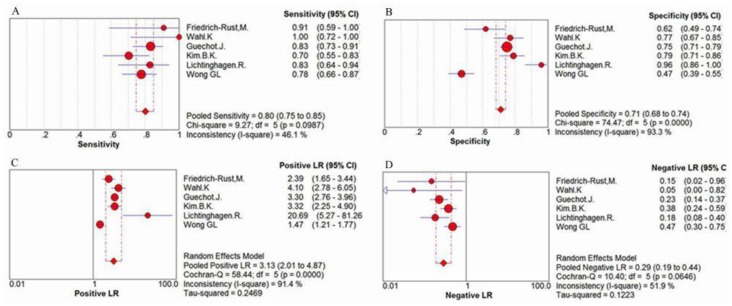
Forest plot and meta-analysis of studies evaluating (A) the sensitivity, (B) the specificity, (C) the positive LR and (D) the negative LR of ELF test versus liver biopsy for the detection of cirrhosis.

**Figure 8:**
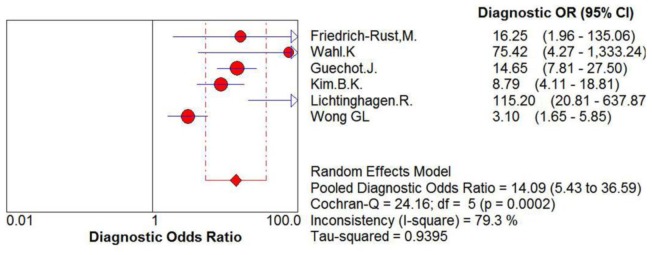
Forest plot and meta-analysis of studies assessing the diagnostic odds ratio of ELF test versus liver biopsy for the prediction of cirrhosis.

For the evaluation of cirrhosis, the sensitivity values of ELF test ranged from 70% to 100%, and the pooled sensitivity value was 80% (95% CI = 0.75–0.85, P = 0.0987, I^2^ = 46.1%). The specificity values ranged from 47% to 95%, and the pooled specificity value was 71% (95% CI: 0.68–0.74, P<0.0001, I^2^ = 99.3%). The pooled positive likelihood ratio was 3.13 (95% CI = 2.01–4.87, P<0.0001, I^2^ = 91.4%), and the pooled negative likelihood ratio was 0.29 (95% CI = 0.19–0.44, P = 0.0646, I^2^ = 51.9%) (Fig. 7) The summary diagnostic odds ratio was 14.09 (95% CI = 5.43–36.59, P = 0.0002, I^2^ = 79.3%) (Fig. 8).

**Figure 9:**
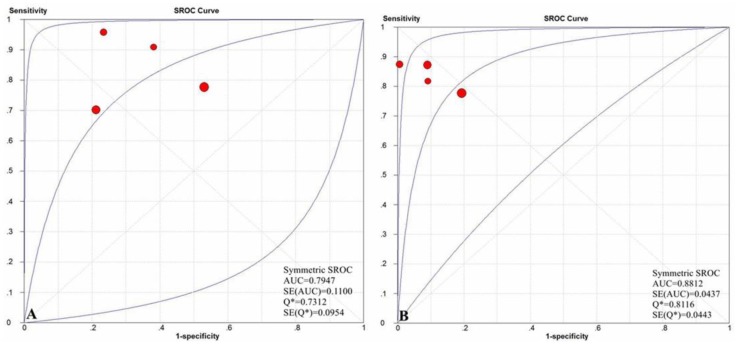
The comparison of SROC curves between (A) ELF test and (B) transient elastogrphy for assessment of cirrhosis.

**Figure 10:**
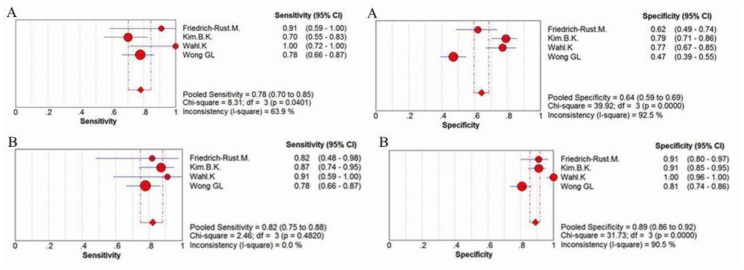
The comparison of sensitivity and specificity between (A) ELF test and (B) transient elastography for assessment of cirrhosis.

**Figure 11:**
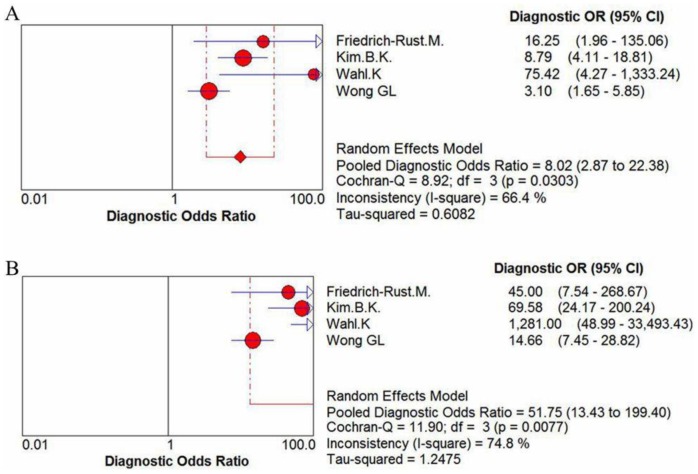
The comparison of diagnostic odds ratio between (A) ELF test and (B) transient elastography for assessment of cirrhosis.

Two [34], [38] of the 4 studies [34], [37], [38], [40] comparing the performance of ELF test with TE for assessment of cirrhosis reported that ELF test has a higher sensitivity, while the all 3 studies [34], [37], [38], [40] uncovered TE has a preferable performance of specificity. More specifically, compared to ELF test that revealed a pooled sensitivity of 78% (95% CI = 0.70–0.85, P = 0.0401, I^2^ = 63.9%), a specificity of 64% (95% CI = 0.59–0.69, P<0.0001, I^2^ = 92.5%) and an AUROC of 0.7947, the transient elastography showed a higher pooled sensitivity 82% (95% CI = 0.75–0.88, P = 0.4820, I^2^ = 0.0%), a higher pooled specificity 89% (95% CI = 0.86–0.92, P<0.0001, I^2^ = 90.5%) and a higher AUROC (0.8812) for detection of cirrhosis(Fig. 9, Fig. 10, Fig. 11).

## Discussion

Information on the presence and degree of liver fibrosis is pivotal for making therapeutic decisions and predicting disease outcomes [41]. For example, the ultimate goal of treatment at the stage of significant liver fibrosis is to prevent the potential pathogenesis of liver disease [41], [42]. In contrast, given that the severe liver fibrosis or cirrhosis may have a risk of progression to hypertension and HCC, discrimination of severe liver fibrosis and cirrhosis is important [42]. The increasing awareness of the limitations of liver biopsies [43], [44] has stimulated the development and refinement of non-invasive techniques for the assessment of liver fibrosis. Theoretically, non-invasive techniques for the assessment of liver fibrosis should possess the advantages of liver specificity, easy execution, and high diagnostic performance, in terms of sensitivity, specificity, DOR, PLR, NLR, and AUROC. The most studied non-invasive detection method for liver fibrosis is transient elastography, but it has shown less accuracy in discriminating lower fibrosis stages [45], [46] and restricted by narrow intercostal space and ascites.

Studies have confirmed that the ELF test can accurately determine the degree of liver fibrosis [32], [47]–[49] and revealed a lower significance for discrimination of low and moderate fibrosis stages and a broad overlapping range for those stages [38]. In the three subgroups of this meta-analysis, the pooled sensitivity, pooled specificity, and summary DOR of the ELF test were greater than 80%, 74%, and 17, respectively. That indicates that at least 74% of patients could reasonably avoid a liver biopsy. With summary AUROCs of 0.8813, 0.8696, and 0.8770 for significant and severe liver fibrosis and cirrhosis, respectively, the results of this meta-analysis demonstrate that ELF has good diagnostic performance for assessing liver fibrosis.

A diagnostic tool is deemed perfect if the AUROC is 100%, excellent if the AUROC is greater than 90%, and good if the AUROC is greater than 80% [50]. According to these results, coupled with its reproducibility, the ELF test can be used in clinical practice as a good tool for the staging of cirrhosis. The fine performance of the ELF test may result from the fact that serum markers reflect fibrosis in the whole liver rather than 1/50,000^th^ of the organ, as does a biopsy sample, or, alternatively, that the ELF test evaluates the impact of liver fibrosis on liver function as well as the architectural damage associated with histological fibrosis and cirrhosis.

It is worth noting that the ELF test showed a high correlation with aminotransferase levels and revealed a significantly high correlation with inflammation [38]. One study [47] found that the ELF test, reflecting on-going pathophysiological processes and functions that a biopsy cannot capture, had prognostic value. The ELF test, an index of HA, PIIINP, and TIMP-1, exhibited prognostic ability even in the early stages of the disease process (AUROC = 0.737–0.863 at all times points) [48], because, probably, the above indices are expressed during the early stages of collagen deposition in the liver. In further analysis of ELF test performance in predicting all-cause mortality, it was found that the AUROC of the ELF test at 6 years was significantly greater than that of a biopsy [47].

In future, the ELF test may be used to evaluate the impact of treatment directed at the underlying causes, such as viral hepatitis, and in the development of new treatments, such as anti-fibrotic drugs.

Indeed, there was significant heterogeneity in this meta-analysis, which may be due to the following reasons. First, differences in study methodologies are well-recognised causes of heterogeneity in meta-analyses of diagnostic tests. Second, subtle variations in the algorithm of the ELF score and liver biopsy may also contribute to between-study variation. Third, the use of different histological scoring systems may result in discrepancies in the findings of the studies. The studies included in this meta-analysis used five histological scoring systems. Although the histological staging system is complex, it is relevant for the assessment, follow-up, and definition of the rate of fibrosis progression, and is also categorical in nature. The current reliance on histological staging using categorical scores for liver biopsy samples is recognised as suboptimal for assessing efficacy, and this may be a source of heterogeneity [51]. Fourth, the size of liver biopsy tissue cores may impact the accuracy of liver fibrosis staging. Criteria for liver biopsy specimens (≥20 mm in length and/or 11 portal tracts) have been described previously [8]. However, in practice, it is difficult for biopsy samples to achieve these criteria. In this meta-analysis, the mean length of specimens ranged from 18.9 to 25.1 mm, so no study reported liver biopsy samples meeting the criteria, and only two studies [32], [36] described liver biopsy specimens with 11 complete portal tracts. Thus, the observed heterogeneity may be secondary to intrinsic errors in liver biopsy measurements, which limit the diagnostic accuracy of non-invasive evaluations [52], [53]. Fifth, a diagnostic threshold (or cut-off value) bias was identified as an important cause of heterogeneity in the pooled results for the three patient groups. In this meta-analysis, there was no consistent cut-off value, which would also generate heterogeneity. Finally, publication bias may also have resulted in heterogeneity in this meta-analysis because we excluded some studies having no full text and published in languages other than English.

In summary, the ELF test showed good performance and considerable diagnostic value for the prediction of histological fibrosis stage and can be deemed a ‘good’ diagnostic tool in clinical practice for the staging of cirrhosis.

## Supporting Information

Checklist S1 The PRISMA Checklist.
(DOC)
